# miR-193a-3p is a potential tumor suppressor in malignant pleural mesothelioma

**DOI:** 10.18632/oncotarget.4346

**Published:** 2015-06-22

**Authors:** Marissa Williams, Michaela B. Kirschner, Yuen Yee Cheng, Jacky Hanh, Jocelyn Weiss, Nancy Mugridge, Casey M. Wright, Anthony Linton, Steven C. Kao, J. James B. Edelman, Michael P. Vallely, Brian C. McCaughan, Wendy Cooper, Sonja Klebe, Ruby C.Y. Lin, Himanshu Brahmbhatt, Jennifer MacDiarmid, Nico van Zandwijk, Glen Reid

**Affiliations:** ^1^ Asbestos Diseases Research Institute (ADRI), Sydney, Australia; ^2^ Sydney Medical School, The University of Sydney, Sydney, Australia; ^3^ Faculty of Pharmacy, The University of Sydney, Sydney, Australia; ^4^ EnGeneIC Ltd., Lane Cove, Australia; ^5^ Department of Medical Oncology, Concord Repatriation General Hospital, Sydney, Australia; ^6^ Department of Medical Oncology, Chris O'Brien Lifehouse, Sydney, Australia; ^7^ Cardiothoracic Surgical Unit, Royal Prince Alfred Hospital, The Baird Institute and Faculty of Medicine, The University of Sydney, Sydney, Australia; ^8^ Australian School of Advanced Medicine, Macquarie University, Sydney, Australia; ^9^ Sydney Cardiothoracic Surgeons, RPA Medical Centre, Sydney, Australia; ^10^ Department of Tissue Pathology and Diagnostic Oncology, Royal Prince Alfred Hospital, Sydney, Australia; ^11^ Department of Anatomical Pathology, Flinders Medical Centre, Adelaide, Australia; ^12^ School of Biotechnology and Biomolecular Sciences, University of New South Wales, Sydney, Australia; ^13^ Division of Thoracic Surgery, University Hospital Zurich, Zurich, Switzerland

**Keywords:** tumor suppressor, mesothelioma, microRNA, miR-193a

## Abstract

Malignant pleural mesothelioma (MPM) is an asbestos-induced cancer with poor prognosis that displays characteristic alterations in microRNA expression. Recently it was reported that the expression of a subset of microRNAs can distinguish between MPM and adenocarcinoma of the lung. However, the functional importance of these changes has yet to be investigated. We compared expression of miR-192, miR-193a-3p and the miR-200 family in normal pleura and MPM tumor specimens and found a statistically significant reduction in the levels of miR-193a-3p (3.1-fold) and miR-192 (2.8-fold) in MPM. Transfection of MPM cells with a miR-193a-3p mimic resulted in inhibition of growth and an induction of apoptosis and necrosis *in vitro*. The growth inhibitory effects of miR-193a-3p were associated with a decrease in MCL1 expression and were recapitulated by RNAi-mediated MCL1 silencing. Targeted delivery of miR-193a-3p mimic using EDV minicells inhibited MPM xenograft tumour growth, and was associated with increased apoptosis. In conclusion, miR-193a-3p appears to have importance in the biology of MPM and may represent a target for therapeutic intervention.

## INTRODUCTION

Malignant pleural mesothelioma (MPM), a disease of the serosal surfaces of the thoracic cavity induced by asbestos, exhibits significant changes in gene expression. Genes involved in proliferation and others related to the poor response to therapy are often upregulated in MPM [1–4]. A number of recent studies have demonstrated significant changes in the expression of microRNAs, small noncoding sequences, 20–24 nucleotides in length, that are involved in post-transcriptional gene regulation of the majority of mRNAs. In MPM, as in other cancer types, there is a trend towards global downregulation of microRNA expression, and a number have been shown to act as tumor suppressors which are in many cases predicted to regulate the protein coding genes typically overexpressed in MPM [5–8]. Therefore, identifying downregulated microRNA with tumor suppressor activities offers a new insight into MPM pathogenesis, and may lead to new therapeutic targets for MPM treatment.

The changes in microRNA expression found in MPM have been exploited in the search for new diagnostic [9, 10] and prognostic markers [11–13], and therapeutic targets [8, 14]. Two early reports identified a subset of microRNAs with differential expression in MPM and lung adenocarcinoma. In the study by Gee *et al*, miR-200c-3p, miR-141-3p, miR-200b-3p, miR-200a-5p, miR-429, miR-203 and miR-205 were all found to be expressed at lower levels in MPM than in lung adenocarcinoma [10]. Similarly, the work by Benjamin *et al* [9] found miR-200c-3p, as well as miR-192-5p, to be overexpressed in peripheral lung adenocarcinoma and other carcinomas that metastasize to the pleura. In contrast, miR-193a-3p was expressed at higher levels in MPM. With this combination, the authors devised a scoring system to discriminate MPM from other tumors, and this forms the basis of a clinically used diagnostic test. As the intention was to identify markers to differentiate between MPM and adenocarcinoma, a comparison between MPM and normal mesothelium was not made in either study.

To better understand the role of these microRNAs in MPM, we investigated their expression in MPM and normal mesothelium samples. Of the 7 microRNAs analysed (miR-193a-3p, miR-192-5p, miR-200b, miR-200c, miR-141, miR-203 and miR-205), we found miR-192-5p and miR-193a-3p to be significantly downregulated in tumors. Functional studies suggest that miR-193a-3p has tumor suppressor qualities in MPM cells both *in vitro* and *in vivo*, and that this can potentially be exploited in a therapeutic approach to MPM.

## RESULTS

### Levels of mature miR-193a-3p and miR-192 are reduced in MPM tumors

**Figure 1:**
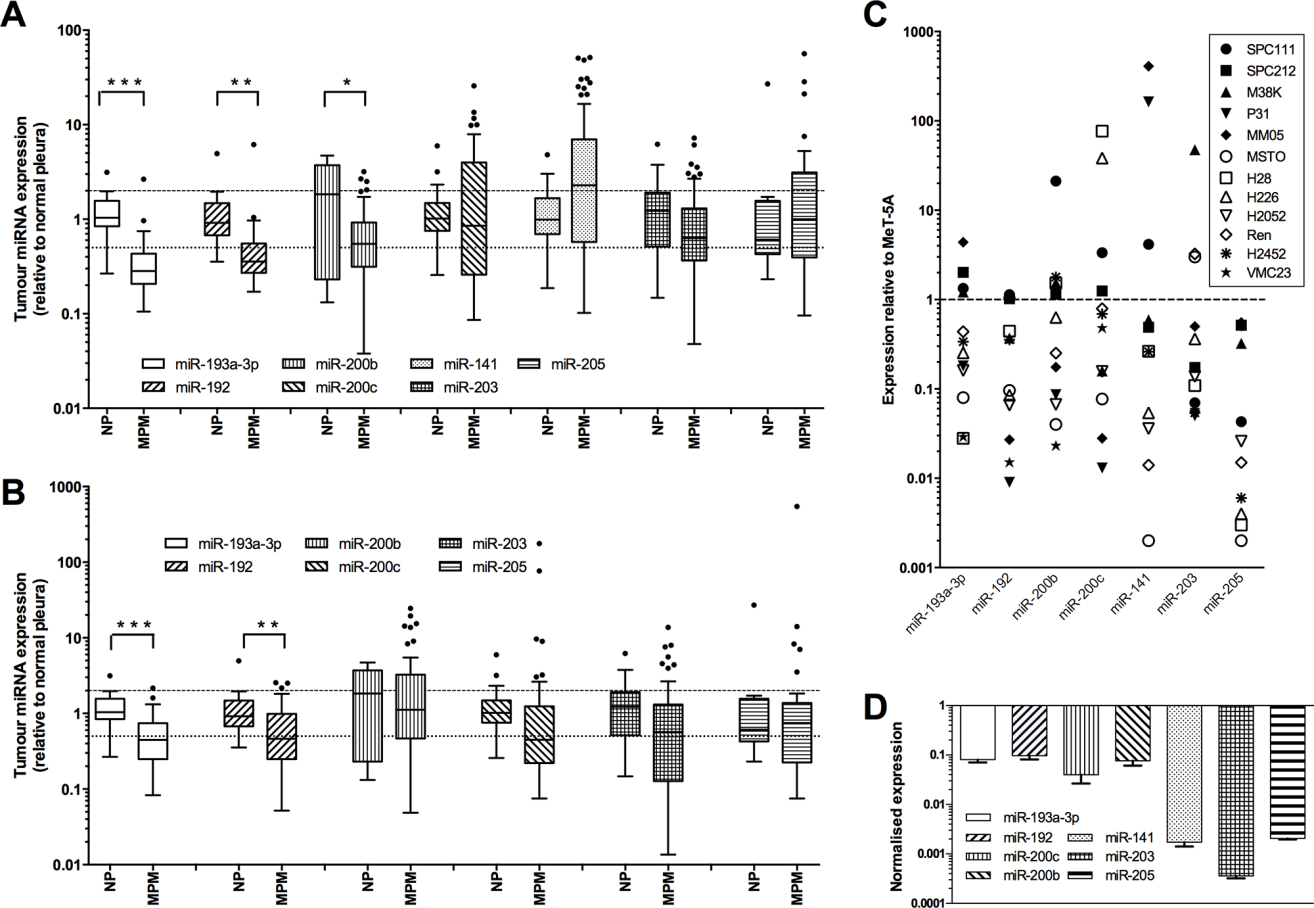
Expression of diagnostic microRNAs is reduced in MPM tumors and cell lines Levels of mature microRNAs were measured in MPM tumor samples from patients undergoing EPP (**A.** ***P = 2.39 × 10^−6^, **P = 0.0007, *P = 0.0034.) or P/D (**B.** ***P = 0.0001, **P = 0.001) and related to levels in normal pleura samples by RT-qPCR, with expression normalized to RNU6B and expressed relative to the average of the controls. Data in A and B are presented as Tukey Box Plot, where the median is represented by the line within the box, and true outliers (>1.5× interquartile range) are represented by the dots outside the boxes. **C.** Expression of individual microRNAs in 12 MPM cell lines was normalized to RNU6B and expressed relative to expression in the immortalized mesothelial line MeT-5A. **D.** Expression of all investigated microRNAs in MSTO cells (normalized to RNU6B and MeT-5A).

Previous studies comparing MPM with adenocarcinomas identified differentially expressed microRNAs with diagnostic value, but the expression of these diagnostic microRNAs has not been assessed in normal mesothelium. We compared the expression of a subset of these diagnostic microRNAs in total RNA isolated from formalin-fixed normal mesothelium and formalin-fixed paraffin-embedded tumor specimens from two patient cohorts [15, 16] using RT-qPCR. In tumor samples from extrapleural pneumonectomy (EPP) patients (Figure 1A), there was significantly reduced expression of both miR-193a-3p (3.1-fold, *P* = 2.39 × 10^−6^) and miR-192 (2.8-fold, *P* = 0.0007) when compared with normal pleura, whereas the reduction in levels of miR-200b (2.3-fold, *P* = 0.0034) and miR-203 (1.5-fold, *P* = 0.1716) was less pronounced. In contrast, miR-200c and miR-141 were not significantly different in tumor and normal samples. Levels of miR-205 were also unchanged, but this microRNA was detected at very low levels and only in a subset of samples (29 of 59 tumors; 8 of 23 normal pleura). Similar results were found in the samples from the pleurectomy ± decortication (P/D) patients; significant downregulation was observed in the expression of miR-193a-3p (2.2-fold, *P* = 0.00001) and miR-192 (2.1-fold, *P* = 0.0001), but there was no significant change in levels of the other microRNAs (Figure 1B). Further analysis of both groups did not reveal a statistically significant difference in microRNA levels between tumors of different histological subtype or stage (data not shown).

### MPM cell lines exhibit a similar downregulation of diagnostic microRNAs

We next analyzed the expression of these diagnostically important microRNAs in a panel of 10 MPM cell lines. The microRNA expression in the MPM cell line panel compared with the control MeT-5A cells is shown in Figure 1C. On average, expression of each microRNA was downregulated in the tumor cells. Most cell lines exhibited reduced expression of at least 4 microRNAs, with the MSTO cells having the most dysregulated expression of this set of microRNAs (Figure 1D). These results reflect the data obtained from tumor samples. As miR-192 is co-transcribed with miR-194–2 (located on chromosome 11) and is closely related to miR-215 (co-transcribed with miR-194-1 on chromosome 1), we investigated whether the expression of these related and co-transcribed microRNAs was similarly reduced, and indeed found that in 3 of 4 cell lines, miR-194 and miR-215 were lower than in normal mesothelial cells (Supplementary Figure 1). The microRNAs with the largest significant downregulation in tumor samples and cell lines, miR-193a-3p and miR-192, were thus selected for further characterization.

### Methylation-induced silencing of MIR193A is not a common event in MPM cells

**Figure 2:**
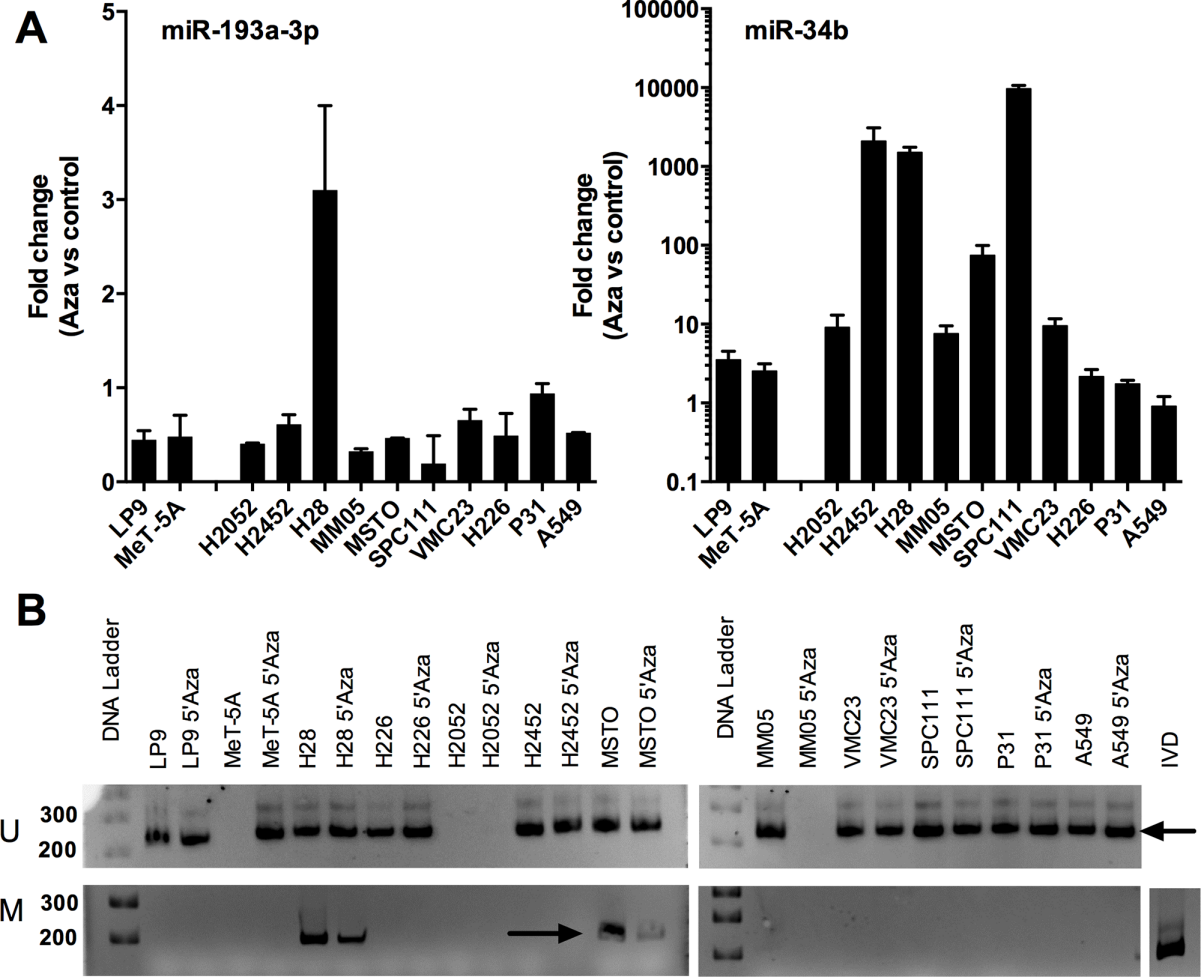
Downregulation of miR-193a-3p is not a result of methylation-induced silencing in MPM cell lines **A.** The expression of miR-193a-3p (**i**) and miR-34c (**ii**) was measured by RT-qPCR in MPM cells after treatment with 5′Aza (5 d) as compared to control. MicroRNA levels were normalized to RNU6B (*n* = 2). **B.** The methylation status of the miR-193a-3p promoter was measured following 5′Aza treatment, showing a band (arrow, upper panels) corresponding to an unmethylated promoter in all untreated and most treated cells. A band (arrow, lower panels) derived from a methylated promoter evident in H28 and MSTO cells reduced in intensity after 5′Aza treatment.

Downregulation of microRNAs in cancer can occur via a number of different mechanisms, and in the case of miR-193a-3p the promoter of the MIR193A gene is associated with CpG islands and is silenced by methylation in lung cancer [17] and AML [18]. To test whether the same mechanism was responsible for the downregulation of miR-193a-3p in MPM, we measured the expression of microRNAs following exposure of cells to the DNA methylation inhibitor decitabine (5-aza-2′-deoxycytidine). This treatment caused a dramatic increase in the levels of miR-34c, a microRNA previously demonstrated to be silenced by methylation in MPM (Figure 2A). Levels of miR-193a-3p were slightly decreased in 9 out of 10 MPM lines, with the one exception the H28 line, in which there was a modest 3-fold increase in miR-193a-3p expression (Figure 2A). Consistent with these findings, there was amplification of a product corresponding to methylated promoter DNA in H28 cells that reduced in intensity following 5′Aza treatment (Figure 2B). Promoter methylation was not detected in MPM lines in which miR-193a-3p levels did not increase, except for MSTO cells, where methylation was detected both before and after 5′Aza treatment without a discernable difference in the expression of miR-193a-3p (Figure 2B). Together, these data suggest that DNA methylation is not the predominant mechanism responsible for the downregulation of MIR193A in MPM cells.

### The effect of miR-192 and miR-193a-3p mimics on the proliferation of MPM cell lines

**Figure 3:**
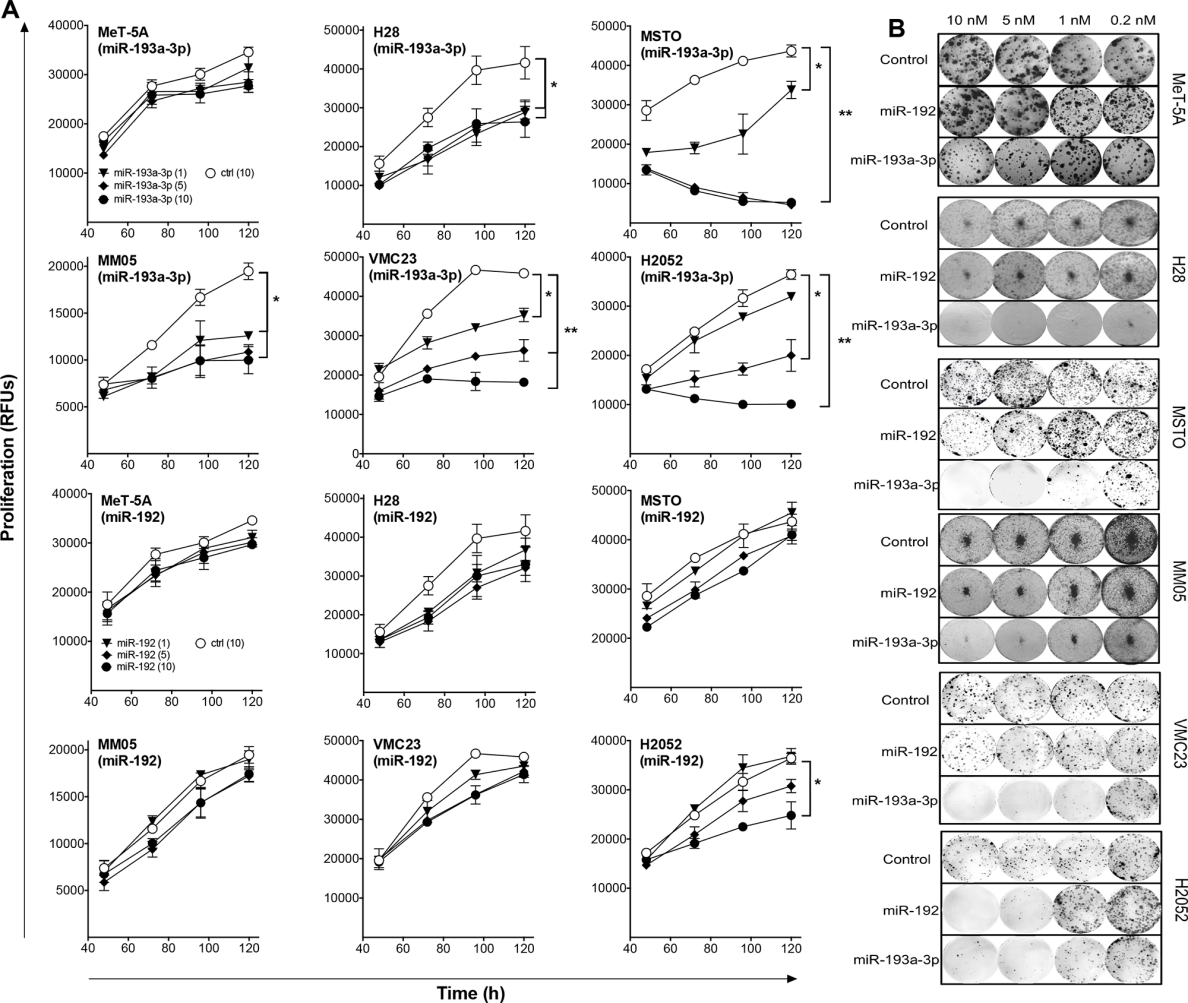
Transfection with miR-193a-3p and miR-192 mimics inhibits growth of MPM cells **A.** Growth of MPM cells was measured after transfection with the indicated concentrations of miR-193a-3p, miR-192 or control mimics. Data are mean ± SD of triplicate measurements and are representative of 3 experiments producing similar results. **B.** Colony formation in MPM cells following mimic transfection. Data are representative of 3 independent experiments. *p < 0.05; **p < 0.01, Mann-Whitney *t*-test.

To determine the functional effects of miR-193a-3p and miR-192, a panel of MPM and normal mesothelial cell lines were transfected with synthetic mimics. Neither mimic had growth inhibitory effects in the immortalized mesothelial cell line MeT-5A (Figure 3A). Compared with a control mimic which did not affect growth (Supplementary Figure 2), increasing levels of miR-193a-3p resulted in a pronounced time- and dose-dependent growth inhibition in all MPM cell lines tested (Figure 3A, Supplementary Figure 3). In contrast, miR-192 had minimal effect on the growth of MPM cell lines, with the exception of a modest 30–50% inhibition of growth in H28, H2052 (Figure 3A) and H2452 cells (Supplementary Figure 3) when transfected at a concentration of 10 nM. When plated at low density, the ability of cells to form colonies was significantly decreased following transfection with miR-193a-3p, and to a lesser extent miR-192, mimics (Figure 3B). In contrast to previous reports of the effects of miR-193a-3p and miR-192 on drug response other tumor types [19–22], neither miR-192 nor miR-193a-3p enhanced sensitivity to gemcitabine, pemetrexed or cisplatin (Supplementary Figure 4). Based on these data, we focused our further efforts on characterization of the effects of the miR-193a-3p mimic on MPM cells.

### Increased miR-193a-3p levels cause target gene downregulation in MPM cells

**Figure 4:**
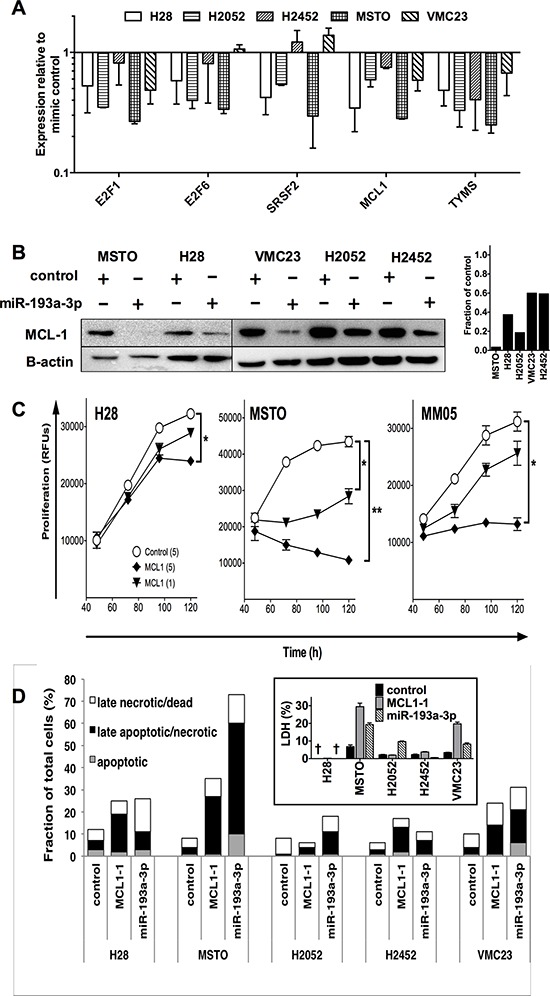
Transfection with miR-193a-3p mimics reduces MCL-1 and increases apoptosis and necrosis in MPM cells **A.** The expression of Mcl-1 and other mRNA targets in MPM cell lines following transfection with miR-193a-3p (5 nM) was measured by RT-qPCR. Data are normalized to RNU6B and control mimic-transfected cells (*n* = 2, mean ± SD). **B.** Expression of MCL-1 protein following miR-193a-3p mimic transfection. Relative MCL-1 expression compared with control mimic transfected cells was determined by densitometry. **C.** Effect of Mcl-1 on MPM cell growth. Cells were transfected with Mcl-1-specific siRNA or control siRNA and proliferation followed over 5 days. Data are representative of 3 independent experiments producing similar results. *p < 0.05; **p < 0.01, Mann-Whitney *t*-test. **D.** MPM cells were transfected with miR-193a-3p, miR-192 or control mimic and after 72 h, apoptotic, late apoptotic/necrotic and late necrotic/dead cell fractions quantified. Data are representative of 3 independent experiments giving similar results. Inset: LDH release from miR-193a-3p or MCL-1 siRNA transfected cells. Values are percentage of maximal LDH released from lysis of an equal number of untransfected cells, and are mean ± SD from triplicate experiments. † = below limit of detection.

To determine whether the growth inhibition seen with miR-193a-3p was associated with downregulation of its previously identified targets, we analyzed expression of selected mRNAs following transfection of MPM cell lines with miR-193a-3p mimics. The mRNA expression of E2F1, E2F6, SRSF2, MCL1 and TYMS were downregulated following transfection with miR-193a-3p (Figure 4A). Levels following transfection with control mimic were similar to untransfected cells (data not shown). On the level of protein expression, MCL-1 was most consistently downregulated in MPM cell lines (Figure 4B), with TYMS and E2F1 also downregulated to a lesser extent (Supplementary Figure 5). In contrast SRSF2 was downregulated in MSTO cells, but upregulated in other MPM lines transfected with miR-193a-3p, and ZEB2, a predicted target of miR-192, remained unaffected by an increase in miR-192 levels (Supplementary Figure 5).

Previous studies have linked the tumor suppressor miR-193a-3p to changes in cell cycle [21], apoptosis [24] and response to cytotoxic drugs [20–22, 25, 26]. Gene enrichment ontology analysis of predicted miR-193a-3p targets revealed that apoptosis was enriched with a *p* value of 0.0001, with additional enriched pathways listed in Table 2. The effects of miR-193a-3p appear to be due, at least in part, to downregulation of the apoptosis-related gene MCL-1, as RNA interference (RNAi)-mediated MCL-1 silencing produced similar effects on growth (Figure 4C). Cell lines exhibiting the greatest growth inhibition following miR-193a-3p (MSTO and MM05) transfection were more inhibited by MCL-1 knockdown than H28 cells, in which the effect of both treatments was more modest. In contrast, cell cycle analysis revealed little change; in the case of MSTO cells, there was an increase in the proportion of cells in the sub-G1 phase, but this was not evident in other MPM cells (Supplementary Figure 6). Analysis of apoptosis and necrosis in the MPM cell lines revealed that the predominant change following transfection with miR-193a-3p mimic or MCL-1 siRNA was an increase in the number of late apoptotic/necrotic cells (Figure 4D). MSTO cells exhibited the greatest increase in late apoptotic/necrotic cells, with over half of the cells labeled with annexin V and PI after levels of miR-193a-3p were increased, and 25% when MCL-1 was silenced. H2052, H2452 and VMC23 showed a more modest increase in the number of late apoptotic/necrotic cells, by 4- to 5-fold following transfection with miR-193a-3p mimic or after MCL-1 knockdown (Figure 4D). An increase in LDH release from MPM cells following transfection with miR-193a-3p mimic or MCL-1 siRNA (Figure 4D inset) further suggests that these treatments induce cell death at least in part by the induction of necrosis.

### Both -3p and -5p arms of miR-193a have growth inhibitory effects in MPM cells

**Figure 5:**
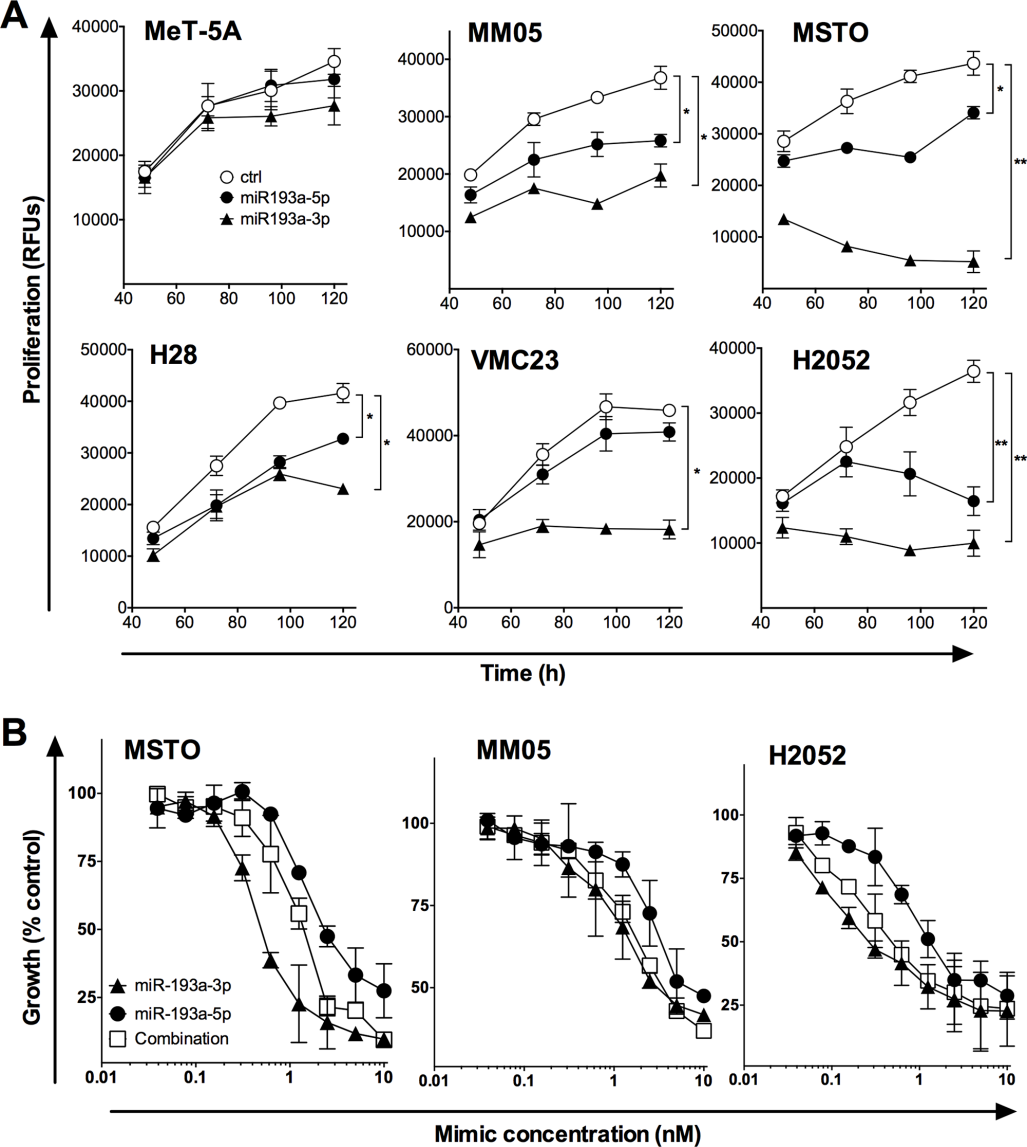
The effect of miR-193a-5p alone and in combination with miR-193a-3p on MPM cell proliferation **A.** MeT-5A and MPM cell lines were transfected with 5 nM miR-193a-3p or miR-193a-5p mimic, or control, and proliferation was followed over 5 days. **B.** MPM cell lines were transfected with the indicated concentration of miR-193a-3p, -5p or a combination, and proliferation measured at 96 h. In both panels, data are representative of 3 independent experiments producing similar results. *p < 0.05; **p < 0.01, Mann-Whitney *t*-test.

In some cases, both the -5p and -3p arms of a pre-miR are found at similar levels and are active in cells, and this appears to be the case for miR-193a-3p and -5p [27]. To determine whether these two active mature microRNAs are coordinately downregulated in MPM, we measured miR-193a-5p levels, and found that it too was detected at significantly lower levels in MPM cell lines compared with normal mesothelial cells (data not shown). In addition, a mimic corresponding to miR-193a-5p was growth inhibitory in MPM cell lines, although to a lesser extent than the miR-193a-3p mimic (Figure 5A). We then attempted to determine whether there was a synergistic effect of combining 5p and 3p mimics, by transfecting both together. Although both mimics were able to inhibit proliferation, transfecting them in combination did not lead to additive effects (Figure 5B).

### Targeted delivery of miR-193a-3p mimic inhibits MPM xenograft tumor growth

**Figure 6:**
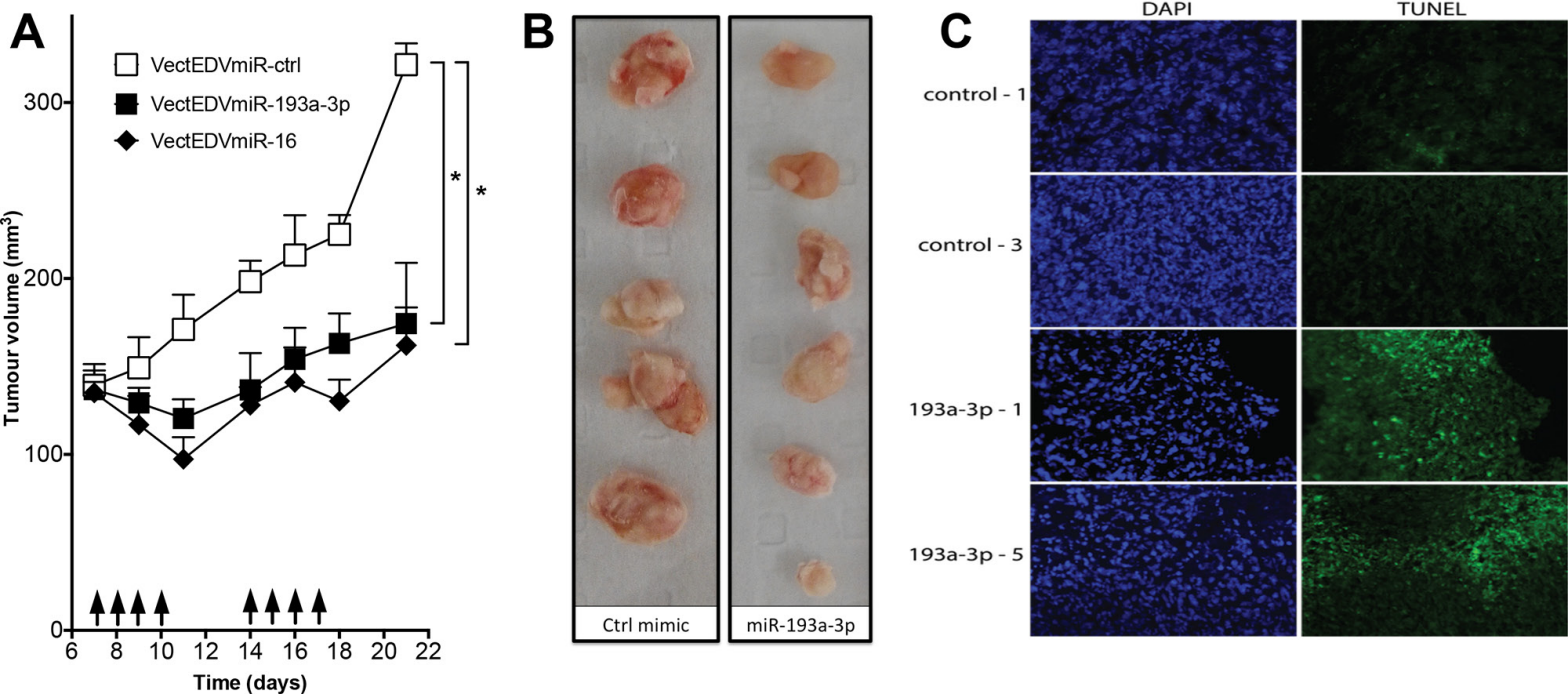
Effect of systemic administration of miR-193a-3p mimics on the growth of MPM xenografts **A.** Growth of MPM xenograft tumors in nude mice upon systemic administration of miR-193a-3p mimic. When tumors reached 150 mm^3^, mice were treated 4 times per week with 2 × 10^9^ EDVs containing miR-193a-3p (*n* = 6), miR-16 (*n* = 6) or mimic control (*n* = 5); *p = 0.015, Wilcoxon signed rank test. **B.** Excised tumors treated with miR-193a-3p mimics were smaller than those treated with controls. **C.** Apoptosis was assessed by TUNEL assay in frozen sections from miR-193a-3p-treated tumors compared with controls. Sections from two representative tumors are shown.

The ability of miR-193a-3p to inhibit tumor growth *in vivo* was investigated in a subcutaneous MPM xenograft model using MSTO-H211 cells implanted in nude mice. Previously we have demonstrated inhibition of tumor growth in this model by restoring levels of the downregulated tumor suppressor microRNA miR-16 delivered using EDV^™^ nanocells (EDVs) [8]. The EDVs are bacterially derived minicells that can be loaded with a variety of cargoes and are delivered via bispecific antibodies [23], and in this case an EGFR-targeted antibody was used. Tumor-bearing mice were treated 4 times per week with EDVs containing miR-193a-3p or miR-16 mimic [8]. The miR-193a-3p-containing EDVs inhibited tumour growth to a similar extent as miR-16 (Figure 6A), resulting in smaller tumours (Figure 6B) with an increase in apoptosis as determined by TUNEL assay (Figure 6C).

## DISCUSSION

Dysregulated microRNA expression is now a well-recognized feature of MPM. Here we show that miR-193a-3p, a microRNA with diagnostic value in MPM, also exhibits tumor suppressor activity in MPM cells. Measuring levels of miR-193a-3p, along with miR-192 and miR-200c, underpins the standardized diagnostic assay marketed as the mirView meso test [9]. In the original study levels of miR-192 and miR-200c were lower, and those of miR-193a-3p higher, in MPM compared with adenocarcinoma. Here we show that expression of both miR-193a-3p and miR-192 is significantly reduced in MPM tumor samples compared with normal pleural tissue, with no change observed in miR-200c levels in the two groups. In this respect, miR-192 downregulation in MPM is similar to that observed for colorectal [28–30] and childhood renal cancer [31], and lower miR-193a-3p reflects findings in oral squamous cell carcinoma [32], acute myeloid leukemia [33], lung cancer [17, 34] and BRAF-mutated melanoma [35]. That we observed no association between miR-193a-3p and clinical outcomes is consistent with a previous report in MPM [13]. Similarly, we did not observe a link between miR-193a-3p expression and histological subtype. Together, this suggests that changes in this microRNA may reflect an early event in the development of mesothelioma.

In addition to miR-192, -193a-3p and -200c, the study by Benjamin *et al* provided data on a further 21 microRNAs differentially expressed between MPM and adenocarcinoma [9]. Of these, the 5 members of the miR-200 family were the most downregulated when comparing levels in MPM with those in adenocarcinoma, and these were also significantly lower in MPM compared with lung carcinoma in the study of Gee *et al* [10]. In contrast to miR-192 and miR-193a-3p, we found little evidence to support a significant difference in expression of the miR-200 family when comparing MPM with samples of normal pleura; only expression of miR-200b was significantly lower in MPM. This is consistent with the findings of Santarelli *et al*, who found no consistent up- or downregulation in these microRNAs, although it should be noted that their study analyzed only a small set of tumors [36].

The study by Gee *et al* also analyzed levels of miR-203 and -205—as together with the miR-200 family these microRNAs are implicated in epithelial-mesenchymal transition (EMT) and control of WNT signalling [10]—and found expression to be significantly lower in MPM than in lung cancer. Although this difference, and a previous study describing lack of miR-203 and miR-429 expression in MPM [6] led the authors to speculate on the role of the entire group of EMT-related microRNAs in MPM biology, these assumptions are only valid if there is a difference in expression levels of these microRNAs between MPM and normal pleura. In our study we observed a reduction in miR-203, although this did not reach significance as levels were extremely variable in both MPM and pleura samples. Similarly, miR-205 levels were very low and frequently undetected in our sample set. Interestingly, in a study of 109 MPM tumors, miR-205 levels were significantly lower in the less differentiated biphasic and sarcomatoid tumors, but again, there was no comparison with normal pleural tissue [37]. Why such a difference was found in miR-205 levels in two studies using similar samples is unclear, but may relate to the different platforms used to detect microRNAs.

The MIR193A gene lies within a CpG island, and previous studies in lung cancer [17, 27, 38], oral squamous cell carcinoma [32] and AML [33] have demonstrated a methylation-induced silencing of the MIR193A gene and corresponding downregulation of miR-193a-3p in tumor cells. Surprisingly, 5′Aza treatment had no effect on levels of miR-193a-3p in the majority of MPM cell lines, but did increase levels of miR-34c, previously shown to be silenced by methylation in MPM [39]. Based on these results, it is tempting to speculate that the frequent methylation of MIR193A in non-small cell lung cancer (NSCLC) [17, 27], but not MPM, is the underlying reason for the observation that expression of miR-193a-3p is lower in NSCLC and other carcinomas than in MPM [9].

As neither deletion of the MIR193A gene nor hypermethylation-induced silencing of its promoter appears to be common in MPM, the downregulation of miR-193a-3p must occur via an alternate mechanism(s). A recent report highlighting the influence of EGFR on microRNA maturation may offer a partial explanation. Under hypoxic conditions, EGFR is internalized into intracellular vesicles, from where it interacts with—and phosphorylates—AGO2 in the cytoplasm [40]. This reduces binding with Dicer, resulting in a dramatic reduction in processing of microRNAs to their mature form. This was especially true for microRNAs with precursors containing long loops, a group to which miR-193a belongs. As MPM tumors are known to frequently exhibit a hypoxic phenotype [4, 41] and often express high levels of EGFR [42, 43], this may explain, at least in part, the reduced levels of mature miR-193a-3p we observe in our tumor series. In addition, maturation of selected primary microRNA transcripts was shown to be augmented by the tumor suppressor BRCA1, which was shown to both recognize RNA secondary structure and associate with DROSHA and DDX5 [44]. Although miR-193a-3p was not included in that study, miR-16 and miR-34a were, and as both are downregulated in MPM without evidence for methylation-induced silencing, the frequent loss of BRCA1 expression in MPM tumors [45] may also be involved in loss of miR-193a-3p.

While previous studies have demonstrated the value of miR-192 and miR-193a-3p in the diagnosis of MPM, our study is the first to provide molecular and functional evidence for a role in MPM biology. The inhibitory effect of miR-193a-3p on MPM growth *in vitro* and *in vivo* is consistent with the inhibition of growth and metastasis in lung cancer [27], induction of apoptosis in glioma [24], acute myeloid leukemia [33] and, to a lesser extent, growth inhibition in hepatocellular carcinoma cell lines [21, 22]. Conversely, miR-193a-3p appears to have the opposite role in bladder cancer [20], where increased levels are associated with growth promotion, similar to the effects of increased miR-193a-5p in squamous cell carcinoma [46]. In terms of the mechanism by which miR-193a-3p mediates growth inhibition, our results in MPM are most comparable with those reported for the glioma cell line U251 [24]. In this line, increasing miR-193a-3p levels produced negligible effects on the cell cycle, but a striking increase in the number of apoptotic cells. Furthermore, in line with our observations in MPM cells, transfection of U251 cells with miR-193a-3p dramatically reduced expression of the anti-apoptotic protein MCL-1, leading to increased evidence of DNA damage and apoptosis; this phenotype was recapitulated in MPM cells by siRNA-mediated MCL-1 knockdown. Taken together, these observations are consistent with the confirmed targeting of MCL-1 3′UTR reporter constructs in glioma [24], ovarian [47] and colorectal [48] cancer cell lines.

MicroRNAs have a well-accepted role in chemoresistance, and in an earlier study we showed that miR-16 is able to sensitize MPM cells to gemcitabine and pemetrexed [8]. In light of reports linking miR-193a-3p and -5p to changes in chemosensitivity, it was therefore surprising that miR-193a-3p did not affect cellular response to drugs commonly used in the clinic to treat MPM. This apparent inconsistency is perhaps explained by context-dependent effects on target genes and mode of action of the drug. For instance, two recent studies revealed seemingly contradictory associations between miR-193a-3p and sensitivity to chemotherapy in hepatocellular carcinoma (HCC). On the one hand, increased miR-193a-3p sensitized HCC cells to the tyrosine kinase inhibitor sorafenib [22], whereas on the other hand, cells resistant to the antimetabolite 5-FU had higher miR-193a-3p expression [21]. An increased level of miR-193a-3p was also found in bladder cancer cells resistant to multiple drugs [20]. In both cases, SRSF2 was downregulated, and the miR-193a-3p-mediated resistance of HCC cells to 5-FU could be reproduced in sensitive cells by miR-193a-3p mimic or SRSF2 siRNA, leading to reduced apoptosis [21]. In MPM cells in contrast, the SRSF2 levels were not consistently altered following transfection with miR-193a-3p mimic. This suggests that the effects of miR-193a-3p on the expression of MCL-1 are the dominant event tipping the balance towards the induction of apoptosis in MPM cells. In the last 5 years, dysregulation of microRNA expression in MPM has been linked to a variety of gene expression changes commonly found in the disease [49]. With advances in design of microRNA mimics and improved delivery strategies, the potential for microRNAs to be used therapeutically is increasing [50]. Previously, we showed that targeted delivery of a miR-16 mimic was able to strongly inhibit tumor growth in a xenograft model of MPM [8]. Using the same EGFR-targeted minicell method for delivery, we have shown here that a miR-193a-3p mimic can exert similar control on MPM tumor growth *in vivo*. While the EGFR expression of the MSTO cell line used in these studies undoubtedly contributes to the success of the minicell treatment, recent studies report EGFR expression in the majority of MPM tumors (membrane immunoreactivity ranging from 68 to 85% [51–53]), suggests that our approach may be broadly translatable to the clinic. Nevertheless, these studies further suggest a lack of EGFR expression in tumors of the sarcomatoid histotype, meaning that alternate antigens will need to be targeted in these cases. Mesothelin represents a good candidate, as it is expressed in virtually all mesotheliomas, and antibodies targeting this protein are the basis of several therapeutic approaches [54].

In conclusion, we have shown that of a series of microRNAs previously shown to have diagnostic value in MPM, two—miR-192 and miR-193a-3p—are downregulated in MPM tumors. Restoring levels of miR-193a-3p led to growth inhibition in MPM cell lines *in vitro* and xenograft tumors *in vivo*. The use of microRNA mimics as an approach to treat cancer is now in early clinical trials [56], and our results suggest that targeted delivery of a mimic of the tumor suppressor microRNA miR-193a-3p, using the same approach we are currently testing in a phase I clinical trial of a miR-15/16-derived mimic (NCT02369198, [57]), may have potential as a therapeutic option for patients with MPM.

## MATERIALS AND METHODS

### Tumor and normal tissue samples

This study used tumor samples from two previously reported series of MPM patients who underwent extrapleural pneumonectomy (EPP) or pleurectomy ± decortication (P/D) at Royal Prince Alfred Hospital (RPAH) or Strathfield Private Hospital (both Sydney, Australia), between 1991 and 2009 (Table 1), and were described previously [15, 58]. This work was approved by the Human Research Ethics Committee (HREC) at Concord Repatriation General Hospital, Sydney, with waiver of consent for the use of samples in this retrospective study (CH62/6/2009/078) granted by the same HREC. Normal pleural tissue (Table 1) was collected as described [8], as part of a study approved by the HREC at RPAH (X10-0342). Written informed consent was obtained from all participants.

**Table 1:** Patient characteristics

	EPP (*N* = 59)	P/D (*N* = 61)	Control (*N* = 23)
Median Age (range)	58 (22–74)	65 (42–83)	66 (53–84)
Sex			
Male	45 (76%)	49 (80%)	19 (83%)
Female	14 (24%)	12 (20%)	4 (17%)
Histology			
Epithelioid	43 (73%)	29 (48%)	
Biphasic	16 (27%)	23 (38%)	
Sarcomatoid	0	9 (15%)	
Pathological^a^ Stage		N/A	
IB	2 (3%)		
II	8 (14%)		
III	44 (75%)		
IV	5 (8%)		

a Pathological stage was determined according to the American Joint Committee on Cancer Staging System [67].

**Table 2:** Enriched pathways among predicted targets of miR-193a-3p

Pathway Name	Enrichment Score	Enrichment *p*-value
Dorso-ventral axis formation	7.76008	0.000426421
GnRH signaling pathway	7.39016	0.000617297
Pathways in cancer	6.60313	0.00135612
Melanogenesis	6.57185	0.0013992
PI3K-Akt signaling pathway	6.15827	0.00211591
Ras signaling pathway	5.7498	0.00318342
Acute myeloid leukemia	5.68478	0.00339728
Renal cell carcinoma	5.49937	0.00408934
Wnt signaling pathway	5.43165	0.00437586
Proteoglycans in cancer	5.01015	0.00666991
MAPK signaling pathway	5.00338	0.00671522
MicroRNAs in cancer	4.88018	0.00759568

Listed are the 12 pathways with an enrichment *P* value of < 0.001.

### Cell lines and cell culture

The MPM cell lines H28, H2052, H2452, H226 and MSTO-211H, the immortalized mesothelial cell line MeT-5A, and the non-small cell lung cancer line A549 were purchased from the American Type Culture Collection (ATCC, Manassas, VA, USA). MM05 [59], VMC23 [60], P31 [61], SPC111 and SPC212 [62] were described previously. LP9 was a gift from Steven Gray (Trinity Centre for Health Sciences, Dublin, Ireland). All cell lines were cultured in RPMI 1640 (MPM lines) or DMEM (MeT-5A, LP9) with 10% fetal bovine serum (FBS) and maintained at 5% CO_2_, 37°C and 95% humidity. All media and FBS were from Life Technologies (Carlsbad, CA, USA). Where indicated, cells were treated with gemcitabine, pemetrexed (Eli Lilly, Sydney, Australia) or cisplatin (McFarlane Medical & Scientific, Sydney, Australia).

### microRNA mimics and transfection

All microRNA mimics were purchased from GenePharma (Shanghai, China) and are listed in Supplementary Table 1. Mimics were reverse transfected into MPM cells using Lipofectamine RNAiMAX (Life Technologies) as previously described [8] and applied at the final concentration indicated in the figures.

### Proliferation and colony formation assays

The effect of microRNA mimics on growth of MPM cell lines was carried out as previously described [8]. Briefly, cells were reverse transfected in 96-well plates using Lipofectamine RNAiMAX (Life Technologies) as described, and following incubation for the indicated time, plates were frozen. Proliferation was measured using a previously described SYBR Green-based assay [63]. Colony formation was assessed after transferring cells transfected in a 96-well plate to 6-well, as described previously [64].

### Apoptosis/necrosis analysis

Apoptosis and necrosis were measured using the Tali Apoptosis Kit (Life Technologies) according to manufacturer's instructions and were analyzed on the Tali Image-Based Cytometer (Life Technologies). Treated samples were normalized to the baseline cell size and Annexin and PI fluorescence parameters of an untreated and unstained cell sample. LDH release was measured as described in the supplement.

### Xenograft studies

The effect on tumor growth of EGFR-targeted EDV^™^ nanocells (EDVs) [23] loaded with miR-193a-3p, miR-16 or control mimic was evaluated in a subcutaneous human xenograft model of MPM in nude mice, as described previously [8], in experiments approved by the EnGeneIC Animal Ethics Committee. Athymic (*nu*/*nu*) mice (4–6 weeks old; purchased from the Animal Resources Centre, Perth, Western Australia) were implanted with 1.5 × 10^6^ MSTO-211H cells in 50 μl serum-free medium mixed with 50 μl growth factor-reduced matrigel (BD Biosciences) via subcutaneous injection in the left flank. Tumor size was determined by measuring length (l) and width (w) and calculating volume (V = lw^2^/2), using measurements obtained by an investigator blinded to the treatment groups. Once the tumor volume reached an average of 150 mm^3^, mice were randomized to different groups before starting the indicated treatments. EDVs were injected systemically via tail-vein injection at the indicated time points.

### TUNEL assay

Apoptotic DNA fragmentation in tumour sections was analysed with the DNA Fragmentation Imaging Kit (Roche Applied Science) following the manufacturer's instructions with slight modification. Briefly, sections were dewaxed (2 × 5 min in xylene), then rehydrated in a graded ethanol series (90%, 80% and 70%, each for 5 min). After washing with PBS, sections were incubated with proteinase K (20 μg/ml) for 30 min at room temperature, washed with PBS, then incubated with terminal deoxynucleotidyl transferase (TdT) in labelled reagent (Bio-16-dUTP) for 60 min at 37°C. A final incubation in SlowFade Gold Antifade Reagent with DAPI was carried out before sections were mounted onto glass slides.

### Real-time RT-qPCR

#### microRNA analysis

Expression of microRNAs was assessed in RNA isolated from two independent series of tumor samples as part of previous studies [8, 12]. For cell lines, RNA was isolated using TRIzol (Life Technologies) as per the manufacturer's instructions, quantified using 260/280 nm readings obtained with a Nanophotometer (Implen, Munich, Germany), then aliquoted and stored at −80°C until further use. RT-qPCR was performed using stem-loop primers for reverse transcription followed by real-time qPCR using hydrolysis probes and primers specific for each microRNA (Life Technologies, see Supplementary Table 1 for TaqMan Assay IDs) as previously described [65] (see Supplementary information for detailed methods). Analysis of global microRNA expression in cell lines made use of TaqMan low-density array cards (Life Technologies), used as per the manufacturer's protocol with 500 ng total RNA as template per card. Array card data were normalized to the reference gene RNU48.

#### mRNA analysis

Cells (1 × 10^6^ per well in 6-well plates) were reverse-transfected with miR-192, miR-193a-3p and control mimics (10 nM) and RNA isolated 48 h post-transfection. Total RNA (500 ng) was reverse transcribed using the Superscript III reverse transcriptase kit (Life Technologies) and amplified with gene-specific primers (Supplementary Table 1) and 2x SYBR Green MasterMix (Life Technologies) according to manufacturer's instructions (detailed in Supplementary Methods).

### Western blot

Cells were reverse transfected with mimics (10 nM) in 10 cm dishes and 72 h post-transfection protein expression was analyzed by Western immunoblot (See Supplementary Methods for details). Most antibodies were diluted in TBST at the following concentrations: SRSF2 rabbit polyclonal (SC35, Abcam, Cambridge, UK) 1:1000, TYMS mouse monoclonal (TS 106, Santa Cruz Biotechnology, Dallas, TX, USA) 1:500, E2F1 mouse monoclonal (KH95, Santa Cruz Biotechnology) 1:100, ZEB2 mouse monoclonal (SIP1, Santa Cruz Biotechnology) 1:500. MCL1 rabbit monoclonal (D35A5, Cell Signaling Technology, Danvers, MA, USA) 1:1000 and beta-actin (AC-15, monoclonal; Sigma-Aldrich, St Louis, MO, USA) 1:1000 were diluted in blocking buffer. The peroxidase-conjugated secondary antibodies used were goat Anti-Rabbit IgG and goat Anti-Mouse IgG (both Thermo Fisher Scientific, Waltham, MA, USA). Quantitative analysis of band size was carried out using densitometry with ImageJ software (v1.48) [66].

### 5′Aza-2′deoxycitidine (5′Aza) treatment

Cells were grown in T25 flasks and at approximately 25% confluence were treated in duplicate with 5′Aza (5 μM) or vehicle (DMSO) every 24 h until conclusion of the assay after 120 h continuous treatment. At this point RNA (from one untreated and one treated flask per cell line) was isolated using TRIzol reagent, and DNA (from the respective second flasks) was isolated using the DNA Mini Kit (Qiagen, Hilden, Germany) with both being performed according to the manufacturer's instructions.

### Methylation-specific PCR (MSP)

MSP was performed as described previously [64] using primers specific for unmethylated and methylated miR-193a-3p promoter. Briefly, 1 μg DNA was bisulfite converted using the Zymo Research DNA Modification Kit (Zymo Research, Orange, CA, USA) with conversion for 18 h at 52°C, and elution in 25 μl elution buffer. MSP for unmethylated (202 bp) and methylated (198 bp) fragments was performed using 2 μl bisulfite-converted DNA as template mixed with 1x AmpliTaq Gold PCR buffer, 250 μM dNTPs, 2mM MgCl_2_, 400 nM forward and reverse primer and 1 unit AmpliTaq Gold DNA polymerase (all Life Technologies). PCR reactions were run on a MultiGene Gradient thermal cycler (Labnet International Inc, Woodbridge, NJ, USA) with 10 min initial denaturing at 95°C followed by 40 cycles of 1 min 95°C denaturing, 1 min 60°C annealing, 1 min 72°C elongation, followed by 10 min final elongation at 72°C. The resultant products were separated on non-denaturing polyacrylamide gels and visualized following ethidium bromide staining using a Kodak GelLogic 2200 imaging system coupled with the Kodak Molecular Imaging software (Integrated Sciences, NSW Australia).

### Pathway analysis

To identify the underlying biological relevance of miR-193a-3p and miR-192 within MPM, we utilized pathway enrichment analysis (Partek Genomics Suite 6.5, Partek Inc, St Louis, MO, USA) of the predicted targets of these microRNAs (TargetScan v6.2). This analysis highlighted gene modules from various regulatory pathways affected by the candidate microRNAs. Gene enrichment analysis using DAVID (Database for Annotation, Visualization, and Integrated Discovery) based on the miR-193 and miR-192 target genes was used to highlight specific categories of genes affected by these microRNAs. These analyses were carried out to highlight the wider impact of miR-193 and miR-192 on the homeostasis of MPM and possibly identify upstream effectors within the regulatory network for potential therapeutic intervention.

### Statistical analysis

Two-tailed independent samples *t*-test was used to compare the differences in microRNA expression between normal pleura and tumor samples. Statistical calculations were done using SPSS version 18.0 for windows (SPSS, Inc., Chicago, IL, USA). A value of *P* < 0.05 was considered statistically significant.

## SUPPLEMENTARY METHODS, TABLE AND FIGURES


