# Supplementary material for: miR-193a-3p is a potential tumor suppressor in malignant pleural mesothelioma
Source: Oncotarget. 2015 Jun 22;6(27):23480–95. doi: 10.18632/oncotarget.4346 (PMC4695131; doi:10.18632/oncotarget.4346)
Supplement: Supplementary file 1 [file oncotarget-06-23480-s001.pdf]

SUPPLEMENTARY METHODS

Cell cycle analysis

Cells were transfected in 6-well plates and 72 h later were fixed with 75% ethanol, refrigerated for a minimum of 24 h, pelleted and resuspended with RNaseA, Propidium Iodide (PI), and 1x PBS (Life Technologies) solution in a 1:12:800 ratio. After incubation at 37°C for 30 min, cells were analyzed on an Accuri C6 flow cytometer (BD Biosciences, Franklin Lakes, NJ, USA) with fluidics set to medium and run with the limit of 15, 000 events. Analysis of flow cytometry data was carried out using FlowJo software version 10 (FlowJo LLC, Ashland, OR, USA).

LDH assay

The release of the intracellular enzyme Lactate Dehydrogenase (LDH) from necrotic cells was measured as described [1]. Cells were reverse transfected in 96-well plates in phenol red-free medium (Life Technologies, Australia) supplemented with 5% FBS (Life Technologies, Australia) and were incubated at 37°C with 5% CO_2 for 72 hours post transfection. Following the incubation period, maximum LDH activity was induced in an equal number of cells in control wells by adding lysis buffer and incubating at 37°C for 45 minutes. Plates were then centrifuged at 250 x g for 5 minutes to remove cell debris and 50 µL of culture supernatant was collected from each well and transferred to a new 96-well plate. To this supernatant, 50 µL of 2 X LDH assay reagent (223 mg 2-p-iodophenyl-3-p-nitrophenyl-5-phenyl tetrazolium chloride, 57 mg N-methylphenazonium methyl sulfate, 575 mg nicotinamide adenine dinucleotide, 3.2 mL lactic acid in 480 mL 200 mM Tris buffer solution, pH 8.0 (Sigma Aldrich, Australia)) was added and the plates were incubated at room temperature in the dark for 30–60 minutes (reaction time was dependent on color development). The reaction was stopped by adding 50 µL stop reagent (1 M HCl) to each well and absorbance was measured between 490 and 520 nm. The percentage LDH release in each sample (corrected for medium only readings) was calculated as a fraction of the maximal LDH released in the control.

RT-qPCR

MicroRNA targets

For reverse transcription we used the TaqMan MicroRNA Reverse Transcription Kit (Life Technologies) with the following reaction conditions: 30 min at 16°C, followed by 30 min at 42°C and 5 min at 85°C. For clinical samples or cell lines, 100 ng total RNA was reverse transcribed in a total reaction volume of 10 µl. All cDNA was used immediately in qPCR reactions or stored at –20°C for use within one week. The qPCR was carried out as per the manufacturer's instructions. Following reverse transcription, as recommended by the manufacturer, the cDNA was further diluted by addition of 28.9 µl in order to achieve a final dilution of 1:15 of the RT product. From the diluted RT product, 2.25 µl were used as template in a triplicate qPCR in a total reaction volume of 10 µl. Amplification was carried out using TaqMan primers/probes specific for each microRNA together with TaqMan 2x Universal PCR MasterMix, No AmpErase UNG (Life Technologies). No template and no RT samples were included as negative controls. Reactions were set up manually and run in triplicate on a ViiA7 Real-Time PCR system with enzyme activation for 10 min at 95°C followed by 40 cycles of 15 s at 95°C and 60 s at 60°C. The Cq (quantification cycle) values were determined applying a fixed threshold of 0.05 using the ViiA7 Software v1.2.2 (Life Technologies). Relative microRNA levels in tumor samples were calculated using a variation of the $2^{-\Delta\Delta Cq}$ method [2] normalized to the reference gene RNU6 and expressed relative to the average ΔCq of all control samples as described previously [3]. In cell lines, data were normalized to RNU6B and expressed relative to levels in MeT-5A cells.

mRNA targets

For analysis of mRNA expression, reverse transcription was carried out on 500 ng RNA using the SuperScript III Reverse transcription Kit in a final reaction volume of 10 µl. In the first step (primer annealing) RNA was incubated for 5 min at 65°C with 2.5 µM Oligo(dT)$_{16–18}$ primer and 500 µM dNTP-Mix. In the second step reaction buffer (final concentration of 1x) was added together with 5 µM DTT, 20 U RNaseOUT and 100 U SuperScript III. Reverse transcription was performed or 60 min at 55°C followed by enzyme inactivation for 15 min at 70°C. The cDNA was diluted 1:10 and 2 µL was used in a 10-µL qPCR reaction with 180 nM each of forward and reverse primers (Supplementary Table 1) and 2x SYBR Green MasterMix (Life Technologies). No-template and no-RT samples were included as negative controls. Reactions were set up manually and run in duplicate on a Viia7 (Life Technologies) instrument with enzyme activation for 10 min at 95°C followed by 40 cycles of 15 s at 95°C and 30 s at 55°C. The Cq values were determined as for microRNAs, with 18S as reference gene.

Western blot

Cell pellets were lyzed with RIPA buffer (25 mM Tris-HCL (pH 7.6), 150 mM, NaCl, 1% (v/v) NP-40, 1% (v/v) Sodium deoxycholate and 0.1% (v/v) SDS) containing protease inhibitors (complete mini EDTA-free protease inhibitor cocktail tablets, Roche Diagnostics Australia), and protein concentration was quantified using the Pierce BCA Protein Assay kit (Thermo Scientific, USA) as per the manufacturer's instructions. Protein (50 µg) was separated on a 10% polyacrylamide gel (Separating: 10% Stock Acrylamide (29:1) 1x Tris-HCl, 0.1% SDS, 0.075% APS, 0.16% TEMED. Stacking: 4% stock Acrylamide, 1x Tris-HCl, pH6.6, 1% SDS, 0.06% APS, 0.46% TEMED) and transferred to Polyvinylidene Fluoride membranes overnight at 40°C using the mini trans-blot central core wet system (Bio-Rad, NSW, Australia). Membranes were blocked with 5% skim milk in TBST (TBS + 0.1% Tween 20, Sigma-Aldrich) for 1 h at room temperature and then probed with target specific antibodies at 4°C overnight. Membranes were washed with TBST and then incubated with a species-specific peroxidase-conjugated secondary antibody diluted 1:1000 in blocking buffer for 1 h at RT (goat Anti-Rabbit IgG for SRSF2 and MCL1, and goat Anti-Mouse IgG for TYMS, E2F1 and ZEB2 (both Thermo Fisher Scientific, USA). Chemiluminescence (Supersignal West Femto Maximum Sensitivity substrate kit, Thermo Fisher Scientific) was used to detect the presence of the protein and was measured using a Kodak Gel Logic 2200 imaging system. Membranes were stripped in BioRad Stripping Reagent for 15 min at room temperature with gentle shaking and washed in TBST before being re-probed for beta-actin as described above. For quantitative analysis by densitometry (using Image J 1.48 software), a box of constant size was placed around each band and density intensities were calculated. Experimental band readings were taken as a percentage of Beta-Actin (control) band readings from the same membrane and results were displayed as a histogram.

REFERENCES

1. Chan FK, Moriwaki K, De Rosa MJ. Detection of necrosis by release of lactate dehydrogenase activity. Methods Mol Biol. 2013; 979:65–70.
2. Livak KJ, Schmittgen TD. Analysis of relative gene expression data using real-time quantitative PCR and the 2(-Delta Delta C(T)) Method. Methods. 2001; 25:402–408.
3. Reid G, Pel ME, Kirschner MB, Cheng YY, Mugridge N, Weiss J, Williams M, Wright C, Edelman JJ, Vallely MP, McCaughan BC, Klebe S, Brahmbhatt H, MacDiarmid JA, van Zandwijk N. Restoring expression of miR-16: a novel approach to therapy for malignant pleural mesothelioma. Annals of oncology : official journal of the European Society for Medical Oncology / ESMO. 2013; 24:3128–3135.

SUPPLEMENTARY TABLE AND FIGURES

Supplementary Table 1: TaqMan assays, primers and mimics used in this study

TaqMan assays

microRNA	TaqMan assay ID	
miR-192-5p	000491	
miR-193a-3p	002250	
miR-200c-3p	002300	
miR-200b-3p	002251	
miR-203-3p	000507	
miR-205-5p	000509	
miR-141-3p	000463	
miR-34c-5p	000428	
miR-15a-5p	000389	
miR-16-5p	000391	
RNU6B	001973	
Primers for RT-qPCR		
Target gene	Forward primer (5′-3′)	Reverse primer (5′-3′)
E2F1	CTGGGTCAACCCCTCAAG	CTGGGTCAACCCCTCAAG
E2F6	ACAAAAGGTCTGAAGGTGTCG	TCAAATGCCATCAGTTGCTT
MCL-1	AAGCCAATGGGCAGGTCT	TGTCCAGTTTCCGAAGCAT
RB1	CTTCCTCATGCTGTTCAGGAG	TGCATGAAGACCGAGTTATAGAAT
SRSF2	AGGTCGCGATCGAAGAGTC	CACTGCTTGCCGATACATCA
TYMS	CCCAGTTTATGGCTTCCAGT	GGTCAACTCCCTGTCCTGAA
WNK1	GGGGTATATCCACCCTCAAGT	GGGAAGATGAAACCCCTGTT
ZEB2	AAGCCTCTGTAGATGGTCCAGT	ACTCCTCGATGCTGACTGC
Primers for methylation studies		
Target gene	Forward primer (5′-3′)	Reverse primer (5′-3′)
Methylated MIR193A	TACGGTTTTCGGTTAGGAGTTC	GATTACCTACGCTCAACTCGAC
Unmethylated MIR193A	GGTTATGGTTTTTGGTTAGGAGTTT	CCTCAATTACCTACACTCAACTCAAC
microRNA mimics		
microRNA	Guide strand (5′-3′)	
miR-192-5p	CUGACCUAUGAAUUGACAGCC	
miR-193a-3p	AACUGGCCUACAAAGUCCCAGU	
miR-193a-5p	UGGGUCUUUGCGGGCGAGAUGA	
miR-control	UUCUCCGAACGUGUCACGUTT	
siRNAs		
Target	Antisense strand (5′-3′)	Sense strand (5′-3′)
Mcl-1-11	GGGAUGGGUUUGUGGAGUUTT	AACUCCACAAACCCAUCCCTT
Mcl-1-12	GCUGGUUUGGCAUAUCUAATT	UUAGAUAUGCCAAACCAGCTT
ctrl	AAGCAACUUGGUAAGACUCGUGUGG	CCACACGAGUCUUACCAAGUUGCUU

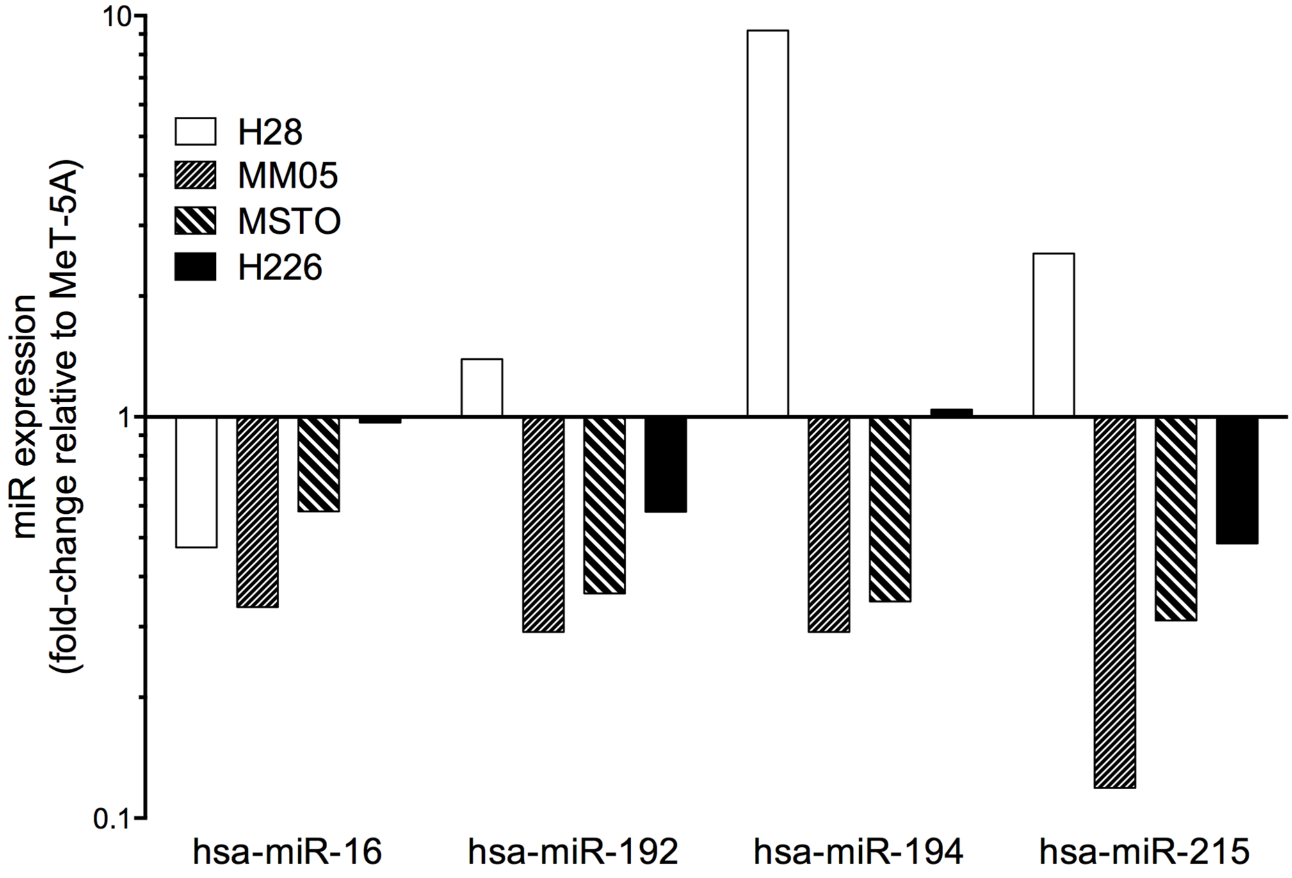

Supplementary Figure 1: Expression of co-transcribed microRNAs in MPM cell lines. Expression of miR-192 and the related miR-194 and miR-215 was measured using TLDA in 4 MPM cell lines and was normalized to RNU6B and expressed relative to expression in the normal mesothelial line MeT-5A. The values for miR-16 are provided for reference, as they represent a previously reported miR downregulated in MPM [3].

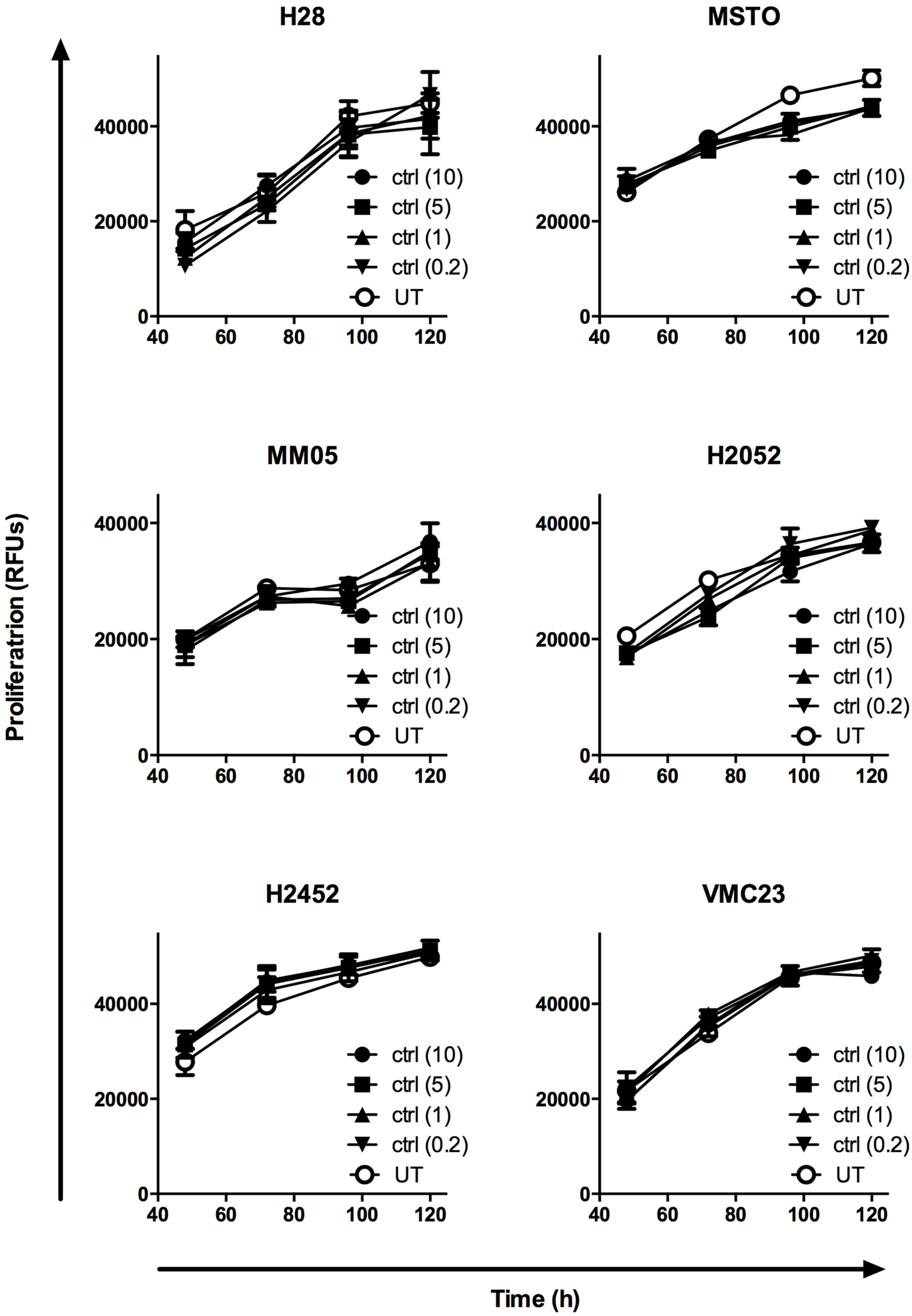

Supplementary Figure 2: Control mimics did not affect growth in MPM cell lines. Growth of the indicated MPM cell lines following transfection with the indicated concentrations of control mimics was compared with untransfected cells. Data are mean ± SD of triplicate measurements and are representative of 3 experiments producing similar results.

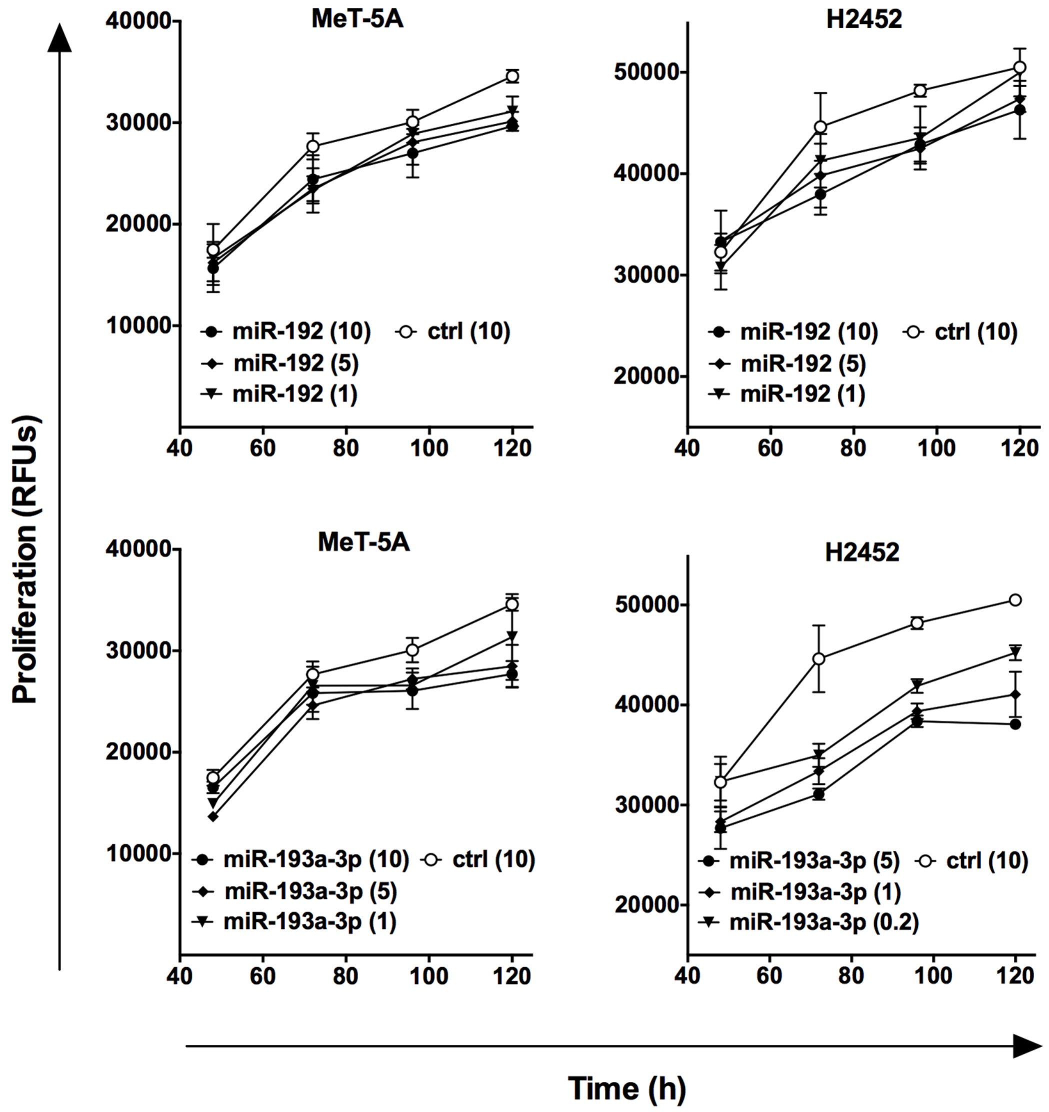

Supplementary Figure 3: Effects of microRNA mimics on growth in additional MPM lines. Growth of the immortalized mesothelial cell line MeT-5A and the MPM cell line H2452 was assessed following transfection with the indicated concentrations of miR-192 (top) or miR-193a-3p (bottom) or control mimics. Data are mean ± SD of triplicate measurements and are representative of 3 experiments producing similar results.

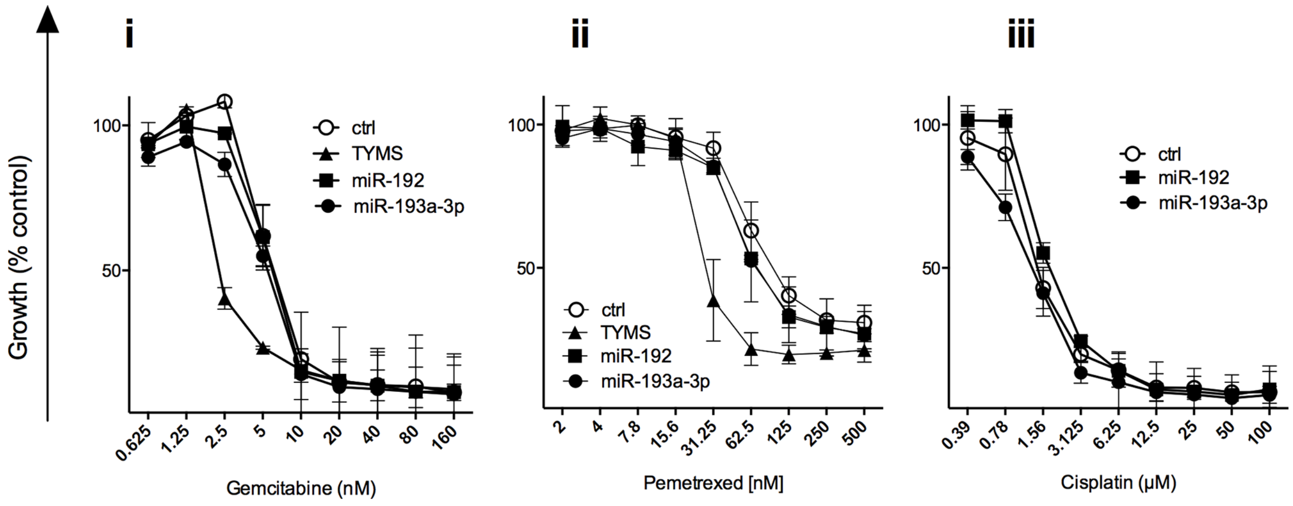

Supplementary Figure 4: Transfection with miR-193a-3p or miR-192 does not affect response to chemotherapeutic drugs. MSTO cells were transfected with miR-192, miR-193a-3p or control mimic, or TYMS siRNA, and one day later treated with gemcitabine **i.** pemetrexed **ii.** or cisplatin **iii.** at the concentrations indicated. Data are mean ± SD of triplicate measurements, and are representative of 3 independent experiments.

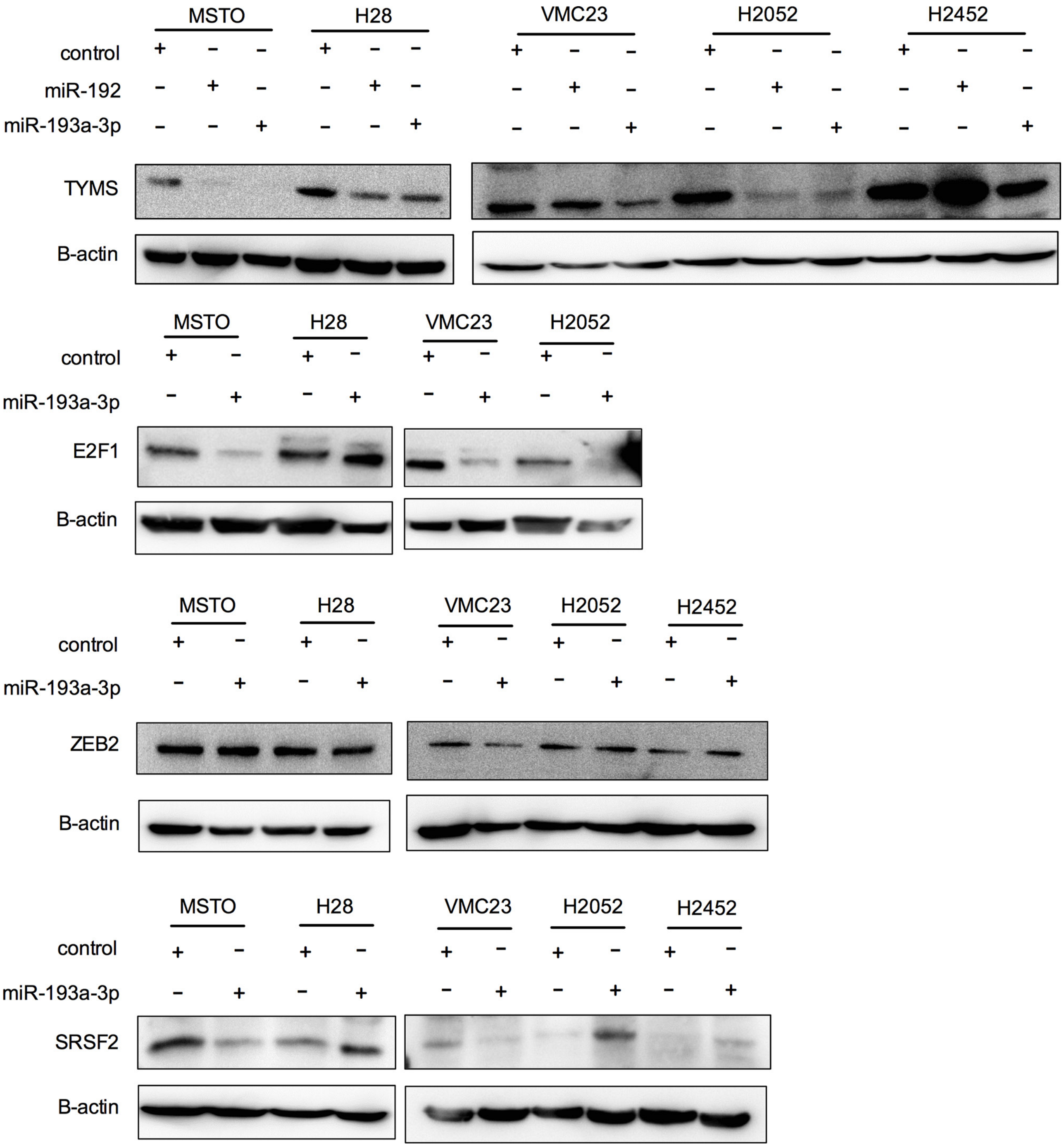

Supplementary Figure 5: Transfection of MPM cell lines with miR-192 or miR-193a-3p mimics does not consistently reduce levels of other targets. Cells were transfected with 10 nM microRNA mimic as indicated, and 48 h later protein isolated. TYMS, E2F1, ZEB2 or SRSF2 were probed, with beta-actin serving as control. Blots are representatives of 3 independent experiments.

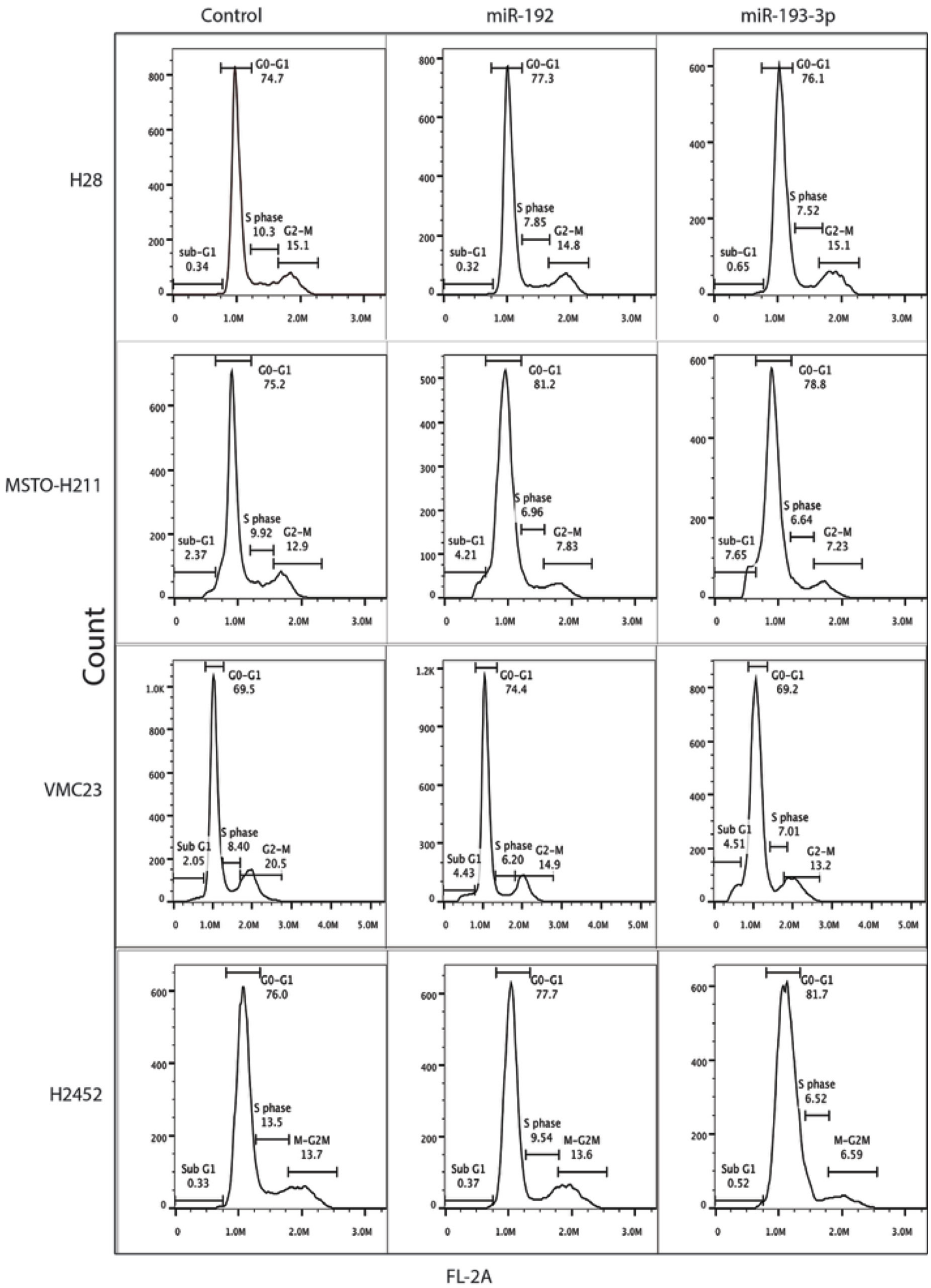

Supplementary Figure 6: miR-193a-3p and miR-192 do not influence cell cycle. MPM cell lines were transfected with the indicated microRNA mimic or control (5 nM) and 48 h later cell cycle distribution was analyzed. Percentage of cells in each compartment is listed in the panels.
